# Mental health of people with distorted body weight perception using medicinal remedies: A representative study

**DOI:** 10.1016/j.ijchp.2021.100224

**Published:** 2021-02-15

**Authors:** Meelim Kim, Seonyeop Kim, Woojun Kim, Hyung Jin Choi

**Affiliations:** a Department of Biomedical Sciences, Seoul National University College of Medicine, Korea; b Department of Medicine, Clinical Counseling Psychology, CHA University, Korea; c 365MC Obesity Clinic, Global 365MC Hospital, Korea; d Neuroscience Research Institute, Wide River Institute of Immunology, Seoul National University, Korea

**Keywords:** Body weight distortion, Weight control strategies, Psychological conditions, Descriptive survey study, Distorsión del peso corporal, Estrategias para control de peso, Condiciones psicológicas, Estudio descriptive mediante encuestas

## Abstract

We aimed to examine the prevalence of distorted body weight perception (BWP) and the choice of weight control strategies to investigate the associations between the psychological features and the different strategies for controlling body weight. Method: We used a representative nationwide 39-item survey to randomly select 1,000 participants. The extrapolated number (eN) to the whole national population was also reported. Self-BWP, weight control strategies, and obesity-related psychological conditions including anxiety, self-esteem, body satisfaction, obesity-related quality-of-life (QoL), and eating attitudes were assessed. Results: Among men, 39.30% (eN = 5,887,137) underestimated, whereas 24.90% (eN = 3,290,847) of women overestimated their weight. In contrast to 2% (eN = 271,745) of men, 15.20% (eN = 2,012,262) of women sought medical support to control their weight. Men and women who used medical support for weight management and women who overestimated their weight reported the most unfavorable psychological conditions (anxiety, self-esteem, body satisfaction, QoL, and eating attitudes; *p* < .05). Conclusions: A prevalent burden of psychological problems related to distorted BWP and weight control strategies was revealed. People with distorted BWP and using medical procedures for their weight control could be at a higher risk of psychological disorders. Therefore, body weight-related psychological problems call for urgent public health policies.

## Antecedentes/Objetivo

Examinar la prevalencia de percepción distorsionada del peso corporal (BWP) y elección de estrategias de control de peso para investigar asociaciones entre características psicológicas y diferentes estrategias para controlar el peso corporal. Método: Encuesta representativa de 39 ítems a nivel nacional para seleccionar al azar a 1,000 participantes. Se informó número extrapolado (eN) a toda la población nacional. Se evaluaron auto-BWP, estrategias de control de peso y condiciones psicológicas relacionadas con obesidad, ansiedad, autoestima, satisfacción corporal, calidad de vida (QoL) relacionada con la obesidad y actitudes alimentarias. Resultados: Entre los hombres, el 39,30% (eN = 5,887,137) subestimó, mientras que el 24,90% (eN = 3,290,847) de mujeres sobreestimó su peso. A diferencia del 2% (eN = 271,745) de los hombres, el 15,20% (eN = 2,012,262) de mujeres buscó apoyo médico para controlar su peso. Hombres y mujeres que utilizaron apoyo médico para el control de peso y mujeres que sobreestimaron su peso informaron condiciones psicológicas más desfavorables (ansiedad, autoestima, satisfacción corporal, calidad de vida y actitudes alimentarias; *p* <  0,05). Conclusiones: Preponderancia de carga de problemas psicológicos relacionadas con BWP distorsionadas y estrategias para el control de peso. Personas con BWP distorsionadas usando procedimientos médicos para el control de peso podrían tener mayor riesgo de trastornos psicológicos. Problemas psicológicos relacionados con peso corporal exigen políticas de salud pública.

People with distorted body weight perception (BWP) have false beliefs that their weight is either above or below their actual body weight. This is problematic since those with distorted BWP tend to initiate inappropriate remedial steps (Kim et al., 2018). Therefore, public health and clinical approaches that can adjust distorted BWP are necessary. Distorted BWP involving overestimation appears more common in women than in men (Park et al., 2019). Previous studies reported women who overestimated their weight as 28.40% among Korean women (Kim et al., 2018), 21% among Chinese women (Niu et al., 2014), 23.50% among American women (Gaylis et al., 2020), and 23.80% among European women (Solmi et al, 2020); in all cases, the proportion of women overestimating their weight was higher than that of men. This trend reflected that women were more affected by the value of being thin, considering it as the ideal body image in the media (Hartman-Munick et al., 2020).

Societal stigma related to obesity is more prevalent in young women and the motivation to manage weight is divergent among age groups (Kim et al., 2018, Pakpour, Chen, et al., 2019, Pakpour, Tsai, et al., 2019). The primary rationale for older adults managing weight was to prevent health risks, whereas for younger adults it was to improve their appearance and engage better in social activities (Robertson et al., 2014).

Weight control strategies (WCS) are determined by BWP (Kim & Seo, 2021). People who underestimate body weight attempt healthy WCS such as healthy diet and exercise while those who overestimate are more likely to pursue unhealthy WCS such as taking diet pills and using laxatives (Park et al., 2019). However, due to WCS categorization difficulties, only few studies have investigated association between BWP and WCS. There are controversial results regarding the role of age and gender in WCS (Frank et al., 2018). In fact, unhealthy WCS serves as serious public health burdens since it is closely related to higher risk for eating disorders, alcohol and tobacco use, and inadequate nutritional intake and quality (Nagata et al., 2018). More in-depth studies are needed to identify the gender and age factors that influence WCS.

Distorted BWP is related to body dissatisfaction and attempting unhealthy WCS, leading to body dysmorphic disorder, health-obsessive behaviors, low quality of life, and eating disorders (Pakpour, Chen, et al., 2019, Pakpour, Tsai, et al., 2019, Park et al., 2019). These adverse symptoms can easily go undetected as the conditions might be disguised by other physical or emotional illnesses (Otakpor & Ehimigbai, 2016). Although mental health conditions related to distorted BWP and unhealthy WCS of the general population requires investigation, previously reported psychological conditions have only focused on specific populations (Kim & Seo, 2021, Pakpour, Chen, et al., 2019, Pakpour, Tsai, et al., 2019, Zarychta et al., 2020). While BWP is known as a multidimensional structure, most previous studies have investigated unidimensional components related to BWP such as quality of life (Cardenas-Lopez et al., 2014, Perdue et al., 2020) or abnormal health-related behaviors (Park et al., 2019). Multidimensional approaches including body shape satisfaction, quality of life, eating attitudes, self-esteem, and trait anxiety would provide a more comprehensive understanding of the negative consequence of BWP. In addition, current WCS especially within medical supports such as bariatric surgery and medications have shown low efficacy and adverse effects in long-term (Kumar and Aronne, 2015, Puzziferri et al., 2014). Therefore, identification of diverse psychological factors corresponding to BWP and WCS is vital to understanding the maladaptive mental health conditions and improve the effectiveness of its treatment.

This study examined the prevalence of BWP and WCS using a nationwide population-based representative sample and the role of age and gender on these characteristics. We assessed the various mental health problems related to both BWP and WCS. This study investigated the mental health condition of people with distorted BWP and WCS, using national representative data. From these objectives, we had the following hypotheses:

Hypothesis 1. Prevalence of distorted BWP and WCS using medical support is greater in women than men and different among the age groups.

Hypothesis 2. Those who have distorted BWP and/or WCS-m might have unfavorable mental health conditions.

## Method

### Participants

This study was a prospective investigation that collected data via a certified online survey system utilizing generalizable population representative over 200,000 panels across South Korea during June 2018 (Open Survey, Seoul, South Korea). This nationwide representative panel was adopted by the Ministry of the Interior and Safety and Korea Agency for Infrastructure Technology Advancement. Inclusion criteria was age between 18 to 49 years old. The exclusion criteria was those who had difficulties using smartphones. A thousand subjects (514 men, 486 women) were randomly selected by the stratified sampling method to adjust the sample group to the whole population with identical distribution of age, gender, and region, allowing extrapolation to the general population. All participants provided informed consent for participation in this research. Ethical approval for the study was obtained from the Institutional Review Board of Seoul National University Hospital.

### Instruments

The survey consisted of 39 items assessing body perception, anxiety, self-esteem, body satisfaction, obesity-related quality-of-life (QoL), eating attitudes, and demographic information. The shortened version of the survey was chosen to reduce the burden of the respondents and maximize the response rate, similar to previous studies (Haugan et al., 2020). All items were selected from reliably validated psychological questionnaires. The items were selected based on the modified items-total correlations and standardized factor loadings which best demonstrated the latent construct and performed low error correlations with other items (Appendix A). The abbreviated scores, derived from the original questionnaires, indicate that the higher scores represent negative psychological states. Therefore, the scores in this study cannot specify whether the person reaches a subclinical or clinical level. The scores should be used as a relative measure, and not an objective diagnostic measure. The reliability of each shortened questionnaire was evaluated with Cronbach’s alpha coefficients. Values greater than .70 were considered acceptable for research purposes (Cicchetti, 1994); alpha coefficients from this study were greater than the defined criterion of .70 except for the Rosenberg Self-Esteem Scale (RSES), which had an alpha coefficient of .63.

To examine the participants’ Body Weight Perception (BWP), they were asked to complete the statement “I perceive my weight status as…” by choosing one out of the four possible responses: “underweight,” “normal,” “overweight,” or “obese.” We compared the participants’ BWP with their actual body mass index (BMI) category and divided their responses into five categories: “highly underestimated,” “underestimated,” “appropriate,” “overestimated,” “highly overestimated.” Highly over/underestimated indicates those who had two stages of discrepancy between the perceived BMI category and actual BMI range. Over/underestimated indicates those with one stage of discrepancy between the perceived BMI category and actual BMI range. For example, “highly overestimated” accounted for those who responded as being “obese” on their weight status although their actual BMI was “normal.” Further, “overestimated” accounted for those who responded as being “overweight” while their actual BMI was “normal.” People in the “highly underestimated” category are those who responded as being “normal” even though their actual BMI was “obese.” Also, “underestimated” presents those who responded as being “normal” while their actual BMI was “overweight.” This assessment has been validated in previous studies (Song et al., 2020). These five categories were rearranged into three categories accordingly: underestimation (combining highly underestimated and underestimated), appropriate estimation (BWP corresponded with their actual BMI), and overestimation (combining overestimated and highly overestimated) for analyzing their association with obesity-related psychological factors.

Weight Control Strategies (WCS) were investigated with the following question, “What strategies do you mostly use to control weight?” with six response options: “exercise,” “dietary,” “diet supplement,” “medication,” “surgery,” or “never attempted.” Their responses were classified into four categories: “exercise,” “dietary,” “medical support (WCS-m),” and “never attempted” for examining their association with obesity-related psychological factors. Diet supplements category was broad and included traditional Chinese medicine, capsules, pills, and liquid, as well as other diet related supplements (Eussen et al., 2011). We considered diet supplements to be a part of the WCS-m category since the survey was conducted as a self-report.

The Korean version of the obesity-related quality-of-life scale (KoQoL). In this study, the KoQoL scale was used to evaluate the participants’ obesity-related QoL. The KoQoL consists of 15 questions and is classified into six areas of mental health, physical health, work, daily life, sexual attraction, and food (Kim & Seo, 2021). Five items were chosen based on the result of the factor analysis, showing higher correlation coefficients, from the previous study (Lee et al., 2013). A higher score indicates a lower QoL. The value of Cronbach’s alphas was .86.

The Eating Attitude Test (EAT-26 K). The EAT-26 K is a 26-item measure of eating attitude. The EAT-26 K has three subscales: identity factors, eating habits, and weight control factors (Barnett et al., 2020). Identity factors indicate abnormal psychological aspects of obesity and eating attitude. Eating habits indicate specific abnormal behavior associated with eating. Weight control factors are related to dietary control. Five items related to the cognitive control of body weight were selected according to the result of reliability test from the Korean version (Jang & Kang, 2017), three items from identity factors and two items from eating habits. A higher score reflects a higher risk of disordered eating attitude. The value of Cronbach’s alphas was .90.

The Body Shape Questionnaire (BSQ-8C). The BSQ-8C is an 8-item measure of body image dissatisfaction. It assesses the frequency of preoccupation with and distress about body size or shape. Four items were chosen based on the results of psychometric testing from the previous study (Pook et al., 2008). The Korean version of BSQ-8C was used, which has been validated in the Korean population and the internal consistency of the BSQ-8C is acceptable. A higher score reflects greater body image concerns. The value of Cronbach’s alphas was .85.

The Rosenberg Self-Esteem Scale (RSES). The RSES is a 10-item measure of self-esteem and we used the Korean version of RSES. Four items were chosen based on the analysis of psychometric properties from the previous study (Wongpakaran & Wongpakaran, 2012). A higher score reflects lower self-esteem. The value of Cronbach’s alphas was .63.

Trait Anxiety Inventory (TAI). The TAI is a 20-item measure of trait anxiety which asks respondents to report how generally they experience anxiety. Three items were extracted based on the result of the psychometric test from the previous study (Iwata et al., 1998). We used the Korean version of TAI, which was validated from the previous study (Kim et al., 2013). A higher score reflects higher trait anxiety. The value of Cronbach’s alphas was .74.

### Statistical analysis

All data were presented as means and SD and were analyzed using SPSS Windows version 25.0 (SPSS Inc., Chicago, IL, USA) and R software (version 3.6.1). Comparison of proportions between sex and other categorical measures was performed using chi-square test. The nature of the differences in psychological measures, according to the status of BWP (“underestimated,” “appropriate,” “overestimated”) and WCS (“dietary,” “exercise,” “WCS-m,” “never attempted”) were tested using ANOVA followed by Fisher’s least significant difference multiple comparison test. Correlations between variables were analyzed using Spearman’s correlation. Binary logistic regression was performed to determine the predictive factors of BWP and WCS by including sex, age, and actual BMI. Significance was defined as *p* < .05.

## Results

### Baseline characteristics of the participants

The demographic characteristics of Koreans who participated in this study are summarized in Table 1. A thousand participants—514 men and 486 women—were analyzed. The cut-off was applied based on the revised Asia-Pacific BMI criteria by the World Health Organization (2000). The prevalence of obesity among men and women was 39.30% and 17.50%, respectively. The highest obesity rate for men and women was among those in their 30 s (47.80%) and 20 s (19.30%), respectively. Actual BMI range distribution was significantly different between men and women (*p* < .001). Furthermore, all age groups showed significant different distributions (*p* < .001) except for the age group 10 s (*p* = .154).

**Table 1:** The actual BMI range of population by gender and age (*N* = 1,000).

	Gender	*N*	Underweight	Normal weight	Overweight	Obese	*p*
Total	Men	514	25 (4.90%)	173 (33.70%)	114 (22.20%)	202 (39.30%)	*p* < .001
	Women	468	53 (10.90%)	273 (56.20%)	75 (15.40%)	85 (17.50%)	
10s	Men	96	18 (18.80%)	42 (43.80%)	14 (14.60%)	22 (22.90%)	*p* = .154
	Women	89	16 (18%)	52 (58.40%)	10 (11.20%)	11 (12.40%)	
20s	Men	129	4 (3.10%)	54 (41.90%)	27 (20.90%)	44 (34.10%)	*p* < .001
	Women	114	21 (18.40%)	54 (47.40%)	17 (14.90%)	22 (19.30%)	
30s	Men	134	1 (0.70%)	39 (29.10%)	30 (22.40%)	64 (47.80%)	*p* < .001
	Women	130	8 (6.20%)	78 (60%)	19 (14.60%)	25 (19.20%)	
40s	Men	155	2 (1.30%)	38 (24.50%)	43 (27.70%)	72 (46.50%)	*p* < .001
	Women	153	8 (5.20%)	89 (58.20%)	29 (19%)	27 (17.60%)	

### The prevalence of BWP

**Figure 1:**
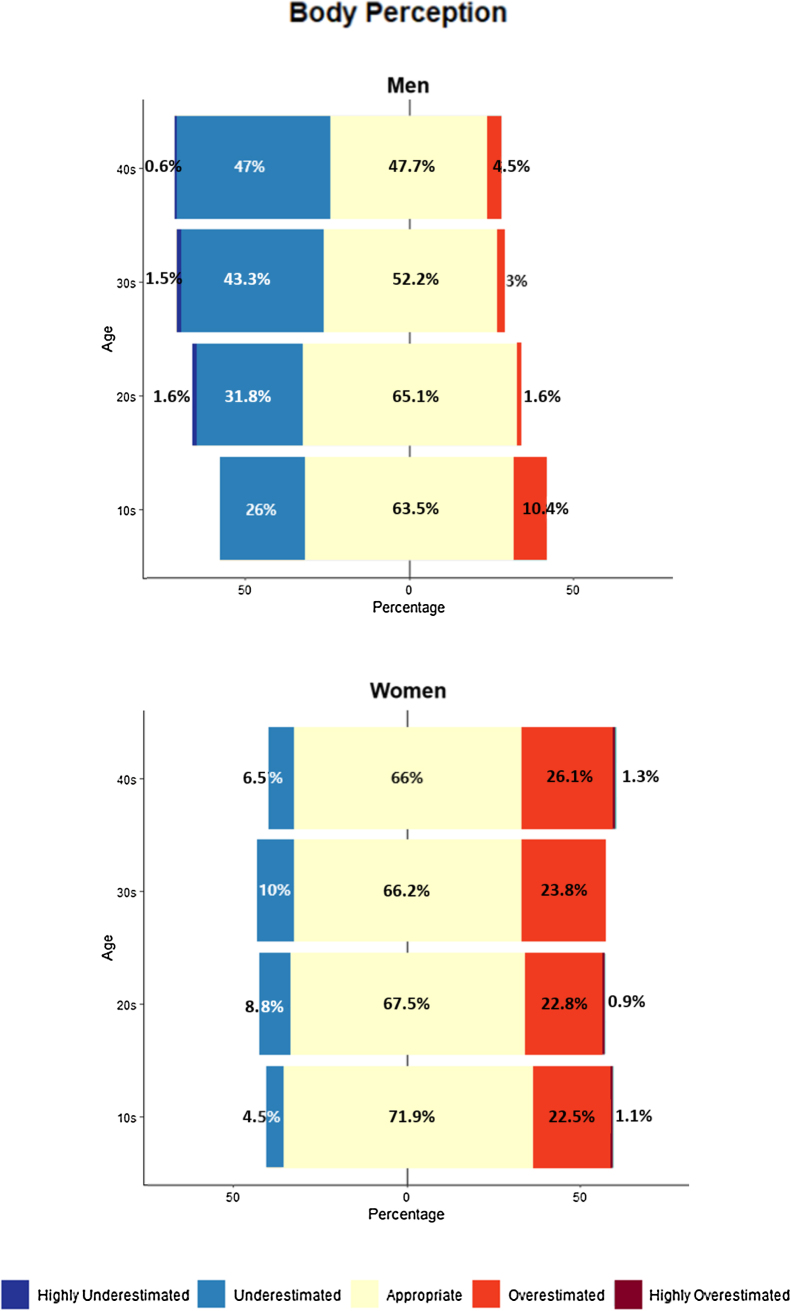
The prevalence of body weight perception.

The prevalence of BWP by sex and age is shown in Figure 1. Among men, 39.30% had underestimated BWP and 24.90% of women had overestimated BWP (*p* < .001). Based on the different age groups, 47.70% of men aged in their 40 s had underestimated BWP and 27.40% of women in their 40 s had overestimated BWP. The ratio of BWP was significantly different by sex (*p* < .001) in all age groups. According to the actual BMI categories, 61.90% and 43.90% of men in obese and overweight groups, respectively, had underestimated BWP while 37.70% and 33% of women in underweight and normal weight groups, respectively, had overestimated BWP. There was a significant difference between sexes in normal weight, overweight, and obese groups (*p* < .001) but there was no significant difference in the underweight group (*p* = .882). Women more likely overestimated BWP; men more likely underestimated BWP. According to binary logistic regression analysis, the risk of overestimated BWP was significantly higher for women (OR 5.34, 95% CI 3.31-8.63) and lower for high actual BMI (OR 0.85, 95% CI 0.80-0.91). The risk of underestimate BWP was significantly higher for men (OR 6.68, 95% CI 4.52-9.88), higher for older age (OR 1.02, 95% CI 1.01-1.05), and higher for high actual BMI (OR 1.12, 95% CI 1.07-1.17).

We estimated the total number of men and women in the Korean population who had distorted BWP as 6,518,572 and 4,302,745, respectively (Table 2). The number of men and women with overestimated BWP was 631,436 and 3,290,847, respectively. The proportion in this cognitive bias group was highest for women in their 40 s (27.40%), and for men in their teens (10.40%). The number of men and women with underestimated BWP was 5,887,137 and 1,011,898, respectively. The men in their 40 s (47.70%) and the women in their 30 s (10%) reported the highest proportions for those who underestimated BWP.

**Table 2:** Prevalence of body weight perception and estimated population.

Gender	Age	Total population (Actual)						
				Highly overestimated	Overestimated	Appropriate	Underestimated	Highly underestimated
Men	10s	2,549,033	%	0	10.40	63.50	26	0
			*n*	-	265,099	1,618,636	662,749	-
	Estimated population per combined category			265,099		1,618,636	662,749	
	20s	3,721,980	%	0	1.6	65.10	31.80	1.60
			*n*	-	59,552	2,423,009	1,183,590	59,552
	Estimated population per combined category			59,552		22,423,009,	21,243,141	
	30s	3,842,450	%	0	3	52.20	43.30	1.50
			*n*	-	115,274	2,005,759	1,663,781	57,637
	Estimated population per combined category			115,274		2,005,759	11,721,418	
	40s	4,255,799	%	0	4.50	47.70	47.10	0.60
			*n*	-	191,511	2,030,016	2,004,481	255,348
	Estimated population per combined category			191,511		2,030,016	2,259,829	
	Overall total			631,436		8,077,420	5,887,137	
Women	10s	2,364,831	%	1.10	22.50	71.90	4.50	0
			*n*	26,013	532,087	1,700,313	106,417	-
	Estimated population per combined category			558,100		1,1,700,31313	106,417	
	20s	3,270,944	%	0.90	22.80	67.50	8.80	0
			*n*	29,438	745,775	2,207,887	287,843	-
	Estimated population per combined category			775,214		2,2,207,8878	287,843	
	30s	3,519,939	%	0	23.80	66.20	10	0
			*n*	-	837,745	2,330,200	351,994	-
	Estimated population per combined category			837,745		2,330,200	351,994	
	40s	4,086,818	%	1.30	26.10	66	6.50	0
			*n*	53,129	1,066,659	2,697,300	265,643	-
	Estimated population per combined category			1,119,788		2,697,300	265,643	
	Overall total			3,290,847		8,935,700	1,011,898	

### The distribution of WCS

**Figure 2:**
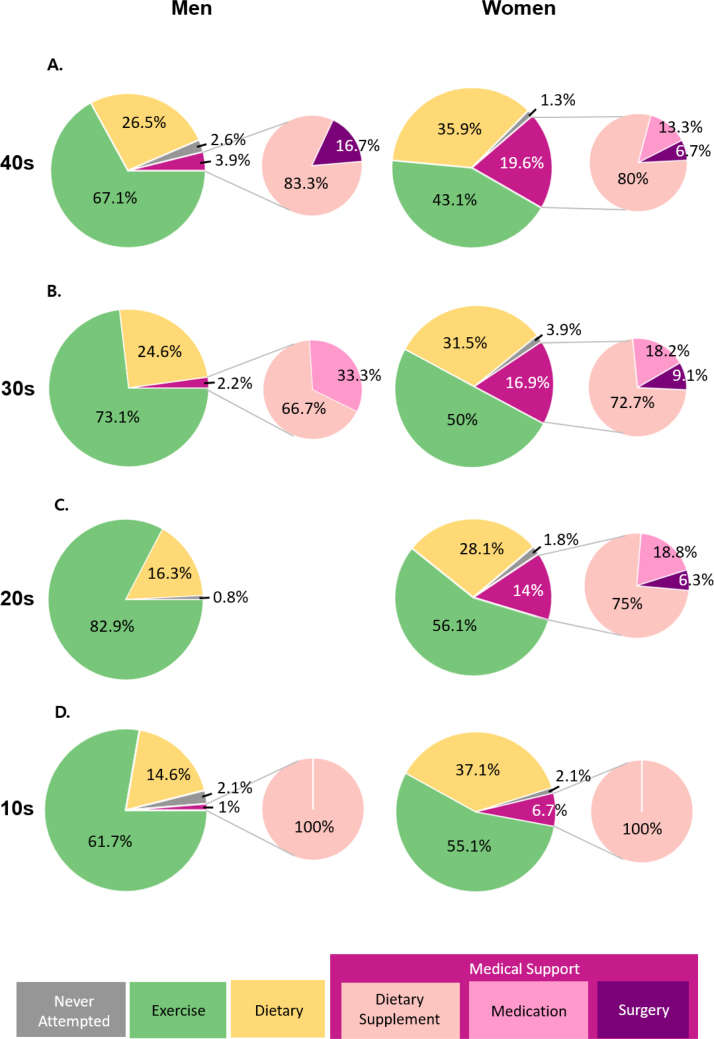
The prevalence of body weight control strategies.

Figure 2 represents the overall distribution of WCS by sex and age. According to the results, 2% of men and 15.20% of women sought out WCS-m. The distribution of WCS were significantly different by sex (*p* < .001). For individuals in their 40 s, 26.50% of men used dietary strategies while 19.60% of women used WCS-m. In the actual BMI categories, 22.80% and 21.90% of men in obese and overweight categories, respectively, attempted to manage their diet while 30.60% and 21.40% of women in the obese and overweight groups, respectively, sought out WCS-m. Among the normal weight, overweight, and obese groups, there was a significant difference for WCS between sexes (*p* < .001). However, in the underweight group, there was no significant difference for WCS between sexes (*p* = .115). Based on the results from binary logistic regression analysis, the risk of using WCS-m was significantly higher for women (OR 14.04, 95% CI 6.89-28.60), older in age (OR 1.04, 95% CI 1.01-1.06), and higher in actual BMI (OR 1.17, 95% CI 1.10-1.25).

The number of men and women who sought WCS-m was 271,745 and 2,012,262, respectively (Table 3). In their 40 s, both men and women reported the highest proportion of using WCS-m at 3.80% and 19.60%, respectively. The highest proportion among the subcategories in WCS-m was dietary supplement, reporting nearly 80% for men and 78.40% for women.

**Table 3:** Prevalence of weight control strategy and estimated population.

Gender	Age	Total population (Actual)							
				Exercise	Dietary	Diet supplement	Medication	Surgery	N/A
Men	10s	2,549,033	%	82.30	14.60	1	0	0	2.1
			*n*	2,097,854	372,159	25,490	-	-	53,530
	Total population per category			2,097,8544	3372,159	25,490			553,5300
	20s	3,721,980	%	82.90	16.30	0	0	0	0.8
			*n*	3,085,521	606,683	-	-	-	29,776
	Total population per category			3,085,521	606,683	0			229,776,7
	30s	3,842,450	%	73.10	24.60	1.50	0.70	0	0
			*n*	2,808,831	945,243	57,637	26,897	-	-
	Total population per category			2,808,831	9945,2434	84,534			0
	40s	4,255,799	%	67.10	26.50	3.20	0	0.6	2.6
			*n*	2,855,641	1,127,787	136,186	-	25,535	110,651
	Total population per category			2,855,641,	1,127,787	161,720			1110,651,
	Overall total			10,847,848	3,051,871	271,745			193,956
Women	10s	2,364,831	%	55.10	37.10	6.70	0	0	1.1
			*n*	1,303,022	877,352	158,444	-	-	26,013
	Total population per category			1,303,022	877,352	158,444			26,013
	20s	3,270,944	%	56.10	28.10	10.50	2.60	0.90	1.8
			*n*	1,835,000	919,135	343,449	85,045	29,438	58,877
	Total population per category			11,835,0000	9919,135,	457,932			58,877
	30s	3,519,939	%	50	31.50	12.30	3.10	1.50	1.5
			*n*	1,759,970	1,108,781	432,952	109,118	52,799	52,799
	Total population per category			11,759,970	1,108,781	594,870			52,799
	40s	4,086,818	%	43.10	35.90	15.70	2.60	1.30	1.3
			*n*	1,761,419	1,467,168	641,630	106,257	53,129	53,129
	Total population per category			1,761,419	1,467,168	1801,016			553,129
	Overall total			6,659,410	4,372,436	2,012,262			190,818

There was a significant difference in the distribution of WCS among BWP (*p* < .001). None of the men with overestimated BWP (*n* = 23) used WCS-m. However, among women with overestimated BWP (*n* = 121), 13.30% (*n* = 16) used WCS-m (9.10% for dietary supplement, 2.50% for medication, and 1.70% for surgery). Among men with appropriate BWP (*n* = 289), 2.30% used WCS-m (1.70% for dietary supplement, 0.3% for medication, 0.30% for surgery). Among women with appropriate BWP (*n* = 328), 16.40% used WCS-m (13.10% for dietary supplement, 2.40% for medication, and 0.90% for surgery). Further, among men with underestimated BWP (*n* = 202), 1.40% used WCS-m (1.40% for diet supplement, and none of them for both medication and surgery). Among women with underestimated BWP (*n* = 37), 10.80% used WCS-m (10.80% for diet supplement, and none of them for both medication and surgery).

**Figure 3:**
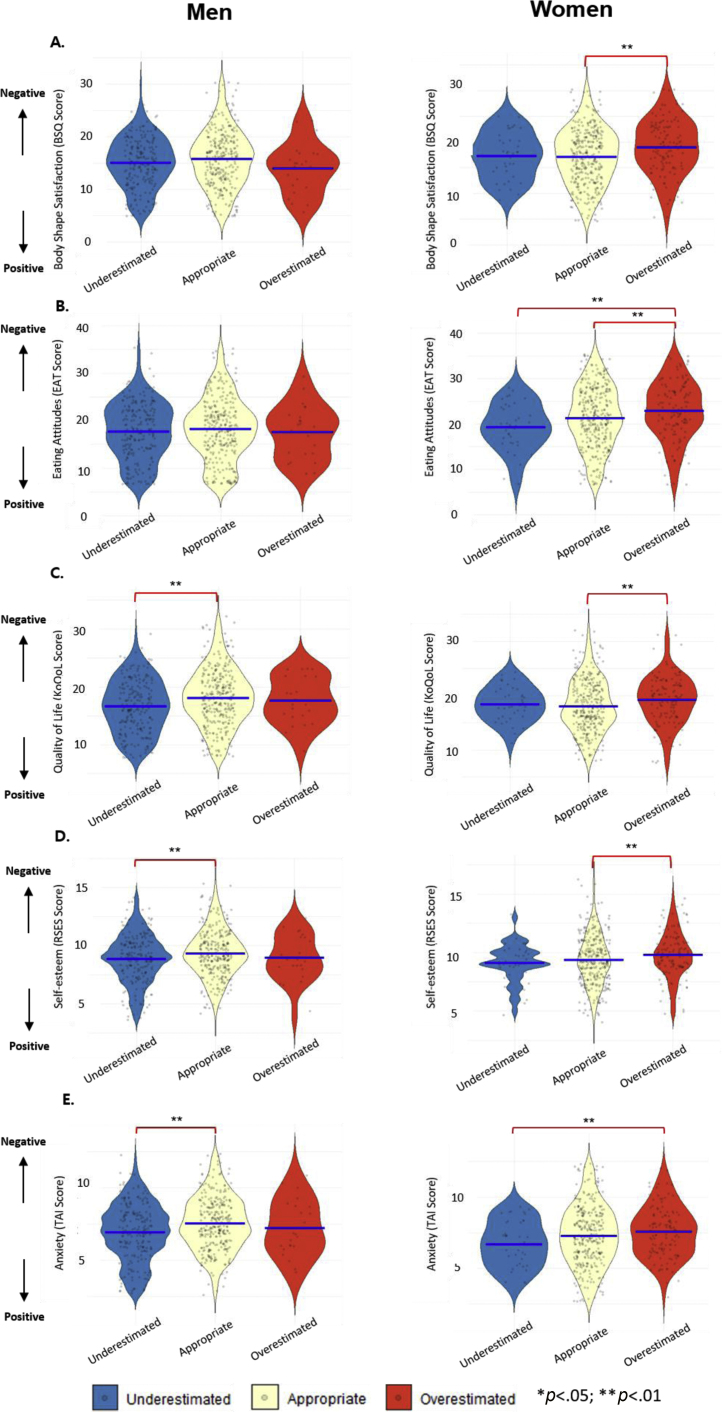
The association between perceived body weight and obesity-related psychological factors.

**Psychological characteristics according to BWP** Figure 3 represents the psychological characteristics according to BWP. Men with underestimated BWP had significantly higher anxiety (*p* < .001), significantly higher self-esteem (*p* = .012), and significantly better KoQoL (*p* = .001) than those with appropriate BWP. In women, compared to people with underestimated BWP, those with overestimated BWP showed significantly lower anxiety (*p* = .021) and had significantly poorer eating attitudes (*p* = .004). Compared to women with appropriate BWP, those with overestimated BWP showed significantly lower self-esteem (*p* = .038) and BSQ-8C (*p* = .001), as well as had significantly poorer eating attitudes (*p* = .027) and KoQoL (*p* = .014).

### Psychological characteristics according to WCS

**Figure 4:**
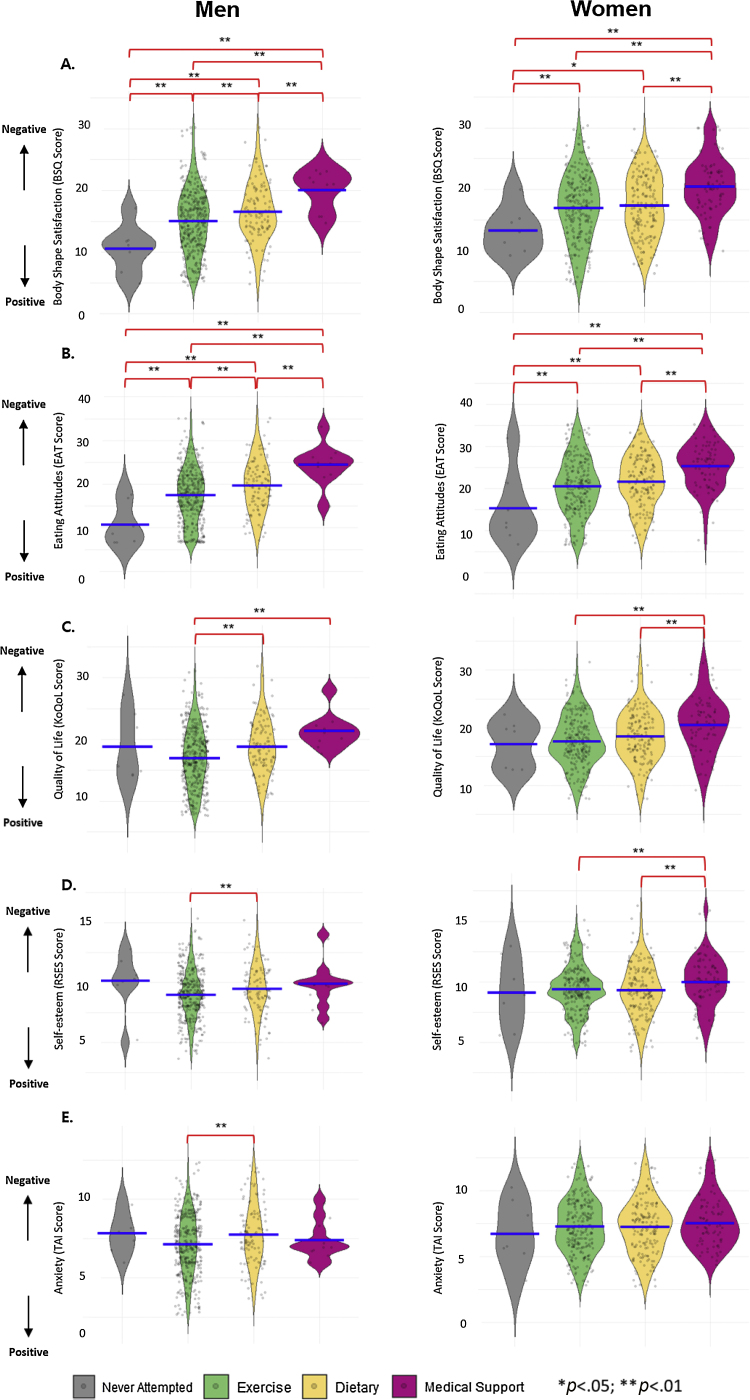
The association between weight control strategies and obesity-related psychological factors.

Figure 4 illustrates the psychological characteristics according to WCS. Among men, those who managed their diet had significantly lower self-esteem (*p* =.025) and KoQoL (*p* < .001) than those who exercised. Men who sought WCS-m showed significantly lower KoQoL (*p* = .004) compared to those who exercised. The BSQ-8C in men who sought WCS-m was also significantly lower than those with dietary control (*p* = .036), exercise (*p* = .002), and no attempt in WCS (*p* < .001). The disordered eating attitudes in men who sought WCS-m was significantly worse than those with dietary control (*p* = .019), exercise (*p* < .001), and no attempt (*p* < .001). The self-esteem of the women seeking WCS-m was significantly lower than those with dietary strategies (*p* = .023) and exercise (*p* = .028). The level of BSQ-8C in women attempting WCS-m was significantly lower than those with dietary control (*p* < .001), exercise (*p* < .001), and no attempt (*p* = .001). The KoQoL in women using WCS-m was significantly lower than those with dietary control (*p* = .002) and exercise (*p* < .001). The disordered eating attitudes in women undertaking WCS-m was significantly worse than those with dietary control (*p* < .001), exercise (*p* < .001), and no attempt (*p* < .001).

## Discussion

In this nationwide-population representative study, 39.30% of men and 7.60% of women underestimated and 4.50% of men and 24.90% of women overestimated their weight. Compared to only 2% of men, 15.20% of women sought WCS-m. Women with overestimated BWP showed the most unfavorable mental health conditions such as higher levels of anxiety and disordered eating attitudes, lower levels of both self-esteem and body shape satisfaction, and poorer condition in obesity-related QoL. Moreover, both men and women who sought WCS-m showed higher level of disordered eating attitudes, lower BSQ, and poorer condition in KoQoL.

This is the first study to report the prevalence of distorted BWP stratified by sex and age using nationwide data (Kim et al., 2018, Park et al., 2019). Greater than expected, 24.90% of women with overestimated BWP relative to their actual BMI proposed that more than three million (3,290,847) women had distorted BWP in the Korean population. The prevalence of underestimated BWP was higher among men than that among women (39.30% vs. 7.60%). Moreover, the prevalence of overestimated BWP among women was higher than that among men (24.90% vs. 4.50%). Similar to our study, the Korea National Health and Nutrition Examination Survey in 2014 found that 28.40% of women overestimated their body weight, higher than men (9.50%) (Kim et al., 2018). However, since the previous study stratified only by sex, it is not feasible to determine what age women would be more likely to suffer from distorted BWP (Abara et al., 2014). Women in their 40 s showed the highest prevalence of distorted BWP toward overestimation and this decreased with age. This result was contradictory to the previous literature reporting a greater overestimation as age decreased (Park et al., 2019). This is due to the difference in methods and the selection bias from low response rates. In cultural aspects, the prevalence of distorted BWP in our study was higher than the previous report in Western countries (Mikolajczyk et al., 2010). Asian women tend to show overestimated BWP compared to Western populations where women underestimating BWP is a more critical issue (Wardle et al., 2006). This can be explained as Asian women tend to compare themselves with slim models in the media and idealizing the idea of a thin body (Martinez-Garcia et al., 2020). Moreover, since Asian cultures are more likely to pursue collectivistic societies than Western cultures, Asian women tend to compare their appearance with others, and are more conscious to others’ judgements (Jung & Lee, 2006), leading to the idealization of thinness.

Distorted BWP can be derived from two different conditions: cognitive-evaluative dysfunction and error in the precision of the body weight (Tovée et al., 2003). The cognitive dysfunction occurs when the person can accurately examine her size, but extremely dissatisfied with her appearance (Gardner, 1996). The error in the precision occurs when the person is unable to assess her body size correctly due to the ignorance and lack of interest (Cash & Deagle, 1997). Using a software system that takes biometric data based on real self-body image and allows individual body parts to be changed freely has been the most realistic approach to determine the cognitive dysfunction related to distorted BWP (Tovée et al., 2003). However, due to the limitation of using a simple survey, the present study could not distinguish between cognitive-evaluative dysfunction or error in the precision of the body weight. Future research focused on distinguishing two different aspects of BWP would yield more accurate results.

This is also the first study to investigate the multi-faceted psychological conditions related to BWP stratified by sex and age with representative data. Our study showed that women with overestimated BWP had a higher degree of body shape dissatisfaction and disordered eating attitudes, poorer QoL, and lower self-esteem. Few studies have only focused on females or adolescents, where they have not examined any more than two psychological aspects (Kim et al., 2018, Martinez-Garcia et al., 2020). A similar study only assessed one psychological symptom, depression, and found that overestimated BWP was positively correlated with depressive symptoms in women (Abara et al., 2014). Another study conducted in China investigated the association between BWP and suggested psychological measures such as anxiety and depression in adolescents samples, showing that girls were more likely to have overestimated BWP and higher levels of depression and anxiety than boys (Xie et al., 2011). Our findings proposed that distorted BWP in women may be a marker of elevated mental health risk. In contrast to limited psychological features in previous reports, this study explored deleterious mental health factors more broadly, such as body shape dissatisfaction, disordered eating attitudes, poor QoL, and low self-esteem. These findings demand urgent public attention and provide cognitive-behavioral therapy (CBT), the most solid empirical support (Wilhelm et al., 2014), toward mental health conditions of women with distorted BWP. Furthermore, annual mental health screening systems for young children and early-aged education related to BWP are needed to prevent the development of distorted BWP.

This study is the first to report the detailed distribution of diverse WCS for sex and age stratifications using nationally representative data. For WCS, 2% of men and 15.20% of women usually sought WCS-m. Specifically, the estimated percentage of women regarding this category was 11.90% for diet supplement, 2.30% for medication, and 1% for surgery in the total population. Women in their 40 s showed the highest proportion adopting WCS-m (19.60%). Using dietary supplements (80%) was the most common method among the subcategories of WCS-m followed by medication (13.30%) and surgery (6.70%) for this population (Figure 2). Comparing our results with previous studies was not feasible since most of the them evaluated the WCS of specific populations with small samples, used fewer categories of WCS, or only gathered information on whether individuals pursued weight control or not (Kim & Seo, 2021, Kim et al., 2018). According to our findings, the major consumers of obesity therapeutics were women (88.10% for WCS-m). Among them, 24.90% had overestimated BWP and only 7.60% had underestimated BWP. Those with overestimated BWP were more likely to seek WCS-m compared to those with underestimated BWP. Therefore, the present study confirmed that the demands for WCS-m are extremely high in Korean women with overestimated BWP.

Despite the society-wide obsession for seeking diverse WCS, little is known about the potential associations between WCS and unfavorable psychological conditions. This study revealed that body shape satisfaction, disordered eating attitude, obesity-related QoL, and self-esteem were significantly lower in women who sought WCS-m than other WCS. To explain these outcomes, a recent study highlighted that people attempting WCS-m may have difficulties adjusting to their new slim body (Perdue et al., 2020). These experiences may negatively influence mental health which could increase the risk of depression and other psychological disorders (Pona et al., 2016). This study clearly demonstrated that these individuals not only experienced depression but also disordered eating attitude, body shape satisfaction, self-esteem, and substandard QoL. Therefore, those who attempted WCS-m would need support to monitor their mental health in obtaining their healthy body image. To reduce the use of WCS-m, it is also suggested to enhance the school-based program and parent training for managing both mental and physical health from early-aged children. In addition, it is recommended to provide mental healthcare services within the use of WCS-m and provide public health programs, allowing easier access to other adaptive weight control strategies (diet and exercise).

To our knowledge, this was the most comprehensive study to examine the association between diverse psychological components and both BWP and WCS using a nationally representative sample in Asia. This also serves as a nationwide report on the prevalence of body shape distortion, with specific categories of WCS and severity of obesity-related psychological welfare in Korea. Importantly, the study data included information about multiple components of obesity-related psychological features. However, there are also limitations to this study. Data were collected using a self-report survey, which could be a bias. Moreover, using the shortened version of the psychological survey would weaken the reliability and the validity of the psychometrics compared to using the full version and has certain risk to make critical bias from the selection of the items. Thus, it is recommended to use full items of each psychological survey and implement objective parameters to reliably explain the mental health state based on the clinical level. Another limitation is that the BWP definition in the present study could not distinguish the concept between cognitive-evaluative dysfunction and error in the precision of the body weight. Thus, especially for assessing BWP, adopting actual body images and video techniques may be the most reliable approach to reduce the bias. Additionally, although the highly overestimated and highly underestimated groups may have more severe psychological problems, the statistical analysis of these extreme groups was not feasible due to the small sample size. Therefore, the future study should involve a larger sample size. Lastly, there is no further investigation for mental health information.

## Conclusions

This nationwide population-study in South Korea clarified that approximately half of men and one third of women had distorted BWP. Among the women, 15.20% sought WCS-m for weight management. Moreover, especially women at age in their 40 s have the highest prevalence of overestimated BWP and WCS-m. These large prevalent populations suffered from multi-dimensional psychological problems. Future studies should undertake screening trials to investigate these psychological issues from early-aged children and conduct longitudinal follow-up studies to observe the psychological factors related to BWP and WCS. Interventions aimed to improve mental health related to distorted BWP and WCS-m are needed to optimize public health. Also, from a nationwide public health perspective, these compelling results call for urgent public health policies to monitor the psychological conditions regarding distorted BWP and WCS-m.

## Funding and acknowledgements

This study was supported by the National Research Foundation of Korea (NRF) Grant funded by the Korean Government [MIST] (No. NRF-2018R1A5A2025964). We would like to thank 365MC Obesity Clinic for supporting this study.

## Appendix A Items chosen for each questionnaire

The Korean version of obesity-related quality-of-life scale (KoQoL): “I feel less confident,” “I want to avoid people,” “I have no confidence in my appearance,” “Exercise makes me suffocate,” “I become lazy and lethargic.”

The Eating Attitude Test (EAT-26 K): “I’m afraid of gaining weight,” “After eating, I feel very guilty,” “I can’t help but think that I’ve gained weight,” “I’m obsessed with food,” “I have an experience of binge.”

The Body Shape Questionnaire (BSQ-8C): “Have you been distracted by your idea of body image?”, “Have you ever thought you were too big and round?”, “Have you ever felt bad about your body image by looking at your reflection?”, “Have you ever been especially conscious of your body image when you were with others?”

The Rosenberg Self-Esteem Scale (RSES): “I don’t have anything to be proud of,” “I think I’m a worthy person or at least similar to others,” “I wish I could be more respectful of myself,” “I tend to take a positive attitude toward myself.”

Trait Anxiety Inventory (TAI): “Difficulties seem to pile up in front of me,” “I tend to be pessimistic,” “I keep coming up with ideas that are not very important.”
